# Dislocations in ceramic electrolytes for solid-state Li batteries

**DOI:** 10.1038/s41598-021-88370-w

**Published:** 2021-04-26

**Authors:** L. Porz, D. Knez, M. Scherer, S. Ganschow, G. Kothleitner, D. Rettenwander

**Affiliations:** 1 FG Nichtmetallisch-Anorganische Werkstoffe, Department of Materials and Earth Sciences, Technical University of Darmstadt, Darmstadt, Germany; 2 Graz Centre for Electron Microscopy, Graz, Austria; 3 Institute of Electron Microscopy and Nanoanalysis, NAWI Graz, Graz University of Technology, Graz, Austria; 4 Leibniz-Institut für Kristallzüchtung (IKZ), Berlin, Germany; 5 Department of Material Science and Engineering, NTNU Norwegian University of Science and Technology, Trondheim, Norway; 6 International Christian Doppler Laboratory for Solid-State Batteries, NTNU Norwegian University of Science and Technology, Trondheim, Norway; 7 Institute of Chemistry and Technology of Materials, NAWI Graz, Graz University of Technology, Graz, Austria

**Keywords:** Chemistry, Energy science and technology, Materials science

## Abstract

High power solid-state Li batteries (SSLB) are hindered by the formation of dendrite-like structures at high current rates. Hence, new design principles are needed to overcome this limitation. By introducing dislocations, we aim to tailor mechanical properties in order to withstand the mechanical stress leading to Li penetration and resulting in a short circuit by a crack-opening mechanism. Such defect engineering, furthermore, appears to enable whisker-like Li metal electrodes for high-rate Li plating. To reach these goals, the challenge of introducing dislocations into ceramic electrolytes needs to be addressed which requires to establish fundamental understanding of the mechanics of dislocations in the particular ceramics. Here we evaluate uniaxial deformation at elevated temperatures as one possible approach to introduce dislocations. By using hot-pressed pellets and single crystals grown by Czochralski method of Li_6.4_La_3_Zr_1.4_Ta_0.6_O_12_ garnets as a model system the plastic deformation by more than 10% is demonstrated. While conclusions on the predominating deformation mechanism remain challenging, analysis of activation energy, activation volume, diffusion creep, and the defect structure potentially point to a deformation mechanism involving dislocations. These parameters allow identification of a process window and are a key step on the road of making dislocations available as a design element for SSLB.

## Introduction

Li-ion batteries (LiBs) are considered as the current key technology towards a clean and efficient transportation for the sustainable development of our society. The energy density of conventional LiBs have reached, however, their limit (~ 250 Wh kg^−1^)^1,2^. Hence, the energy densities are too low to meet the demand of future electric vehicles (> 400 Wh kg^−1^) and other emerging applications^3^. New energy storage concepts, such as solid-state batteries using Li-metal anodes (SSLB) in combination with cathodes having high-capacity (e.g., S), or high-voltage (e.g., layered-structured LiNi_0.8_Co_0.1_Mn_0.1_O_2_ (NCM811) are therefore considered as the next-generation energy storage device delivering energy densities up to 350 Wh kg^−1^ and 500 Wh kg^−1^, respectively^3,4^. One of the most promising candidates to be used as solid electrolyte are sulfides (e.g., Li_6_PS_5_Cl (LPSC)^5,6^)^7,8^, and oxides (e.g., Li_7_La_3_Zr_2_O_12_ (LLZO))^9^ that exhibit room-temperature ionic conductivity rivaling that of liquid electrolytes. However, to reach the promised performance SSLB must reversibly plate a large Li thickness (at least 15 μm or 3 mAh cm^−2^) at a high current rate (> 3 mA cm^−2^) for at least 500 full cycles without the assistance of extreme pressures^10–12^. Today, under such conditions, SSLB invariably fail, and the failure invariably initiates at the interfaces^13–22^. One of the main reasons for battery failure by Li prenetration^23,24^ are current constriction at the interface, so called “hot-spots”, that arise from inhomogeneous electrical field distributions at the Li | solid-electrolyte interface that originates from, e.g., (i) poor contact between Li and the solid electrolyte^25,26^ or (ii) the Li void formation during Li stripping^22,27^. The resulting high local current densities and the high electro-chemo mechanical stresses lead to low Coulombic efficiency, safety hazards, short cycle life, internal short circuits and even catastrophic cell failure^12,28–30^. Hence, homogenizing the current distribution and identifying new methods to plate Li at very high rates is of utmost importance to bring this technology from the lab to the market.

Recently, Janek and co-workers reported the formation of whisker-like Li morphologies and indicated a promotion by screw dislocations that enable very high rates of several hundred mA cm^−2^ avoiding Li penetration^28^. These findings are backed by earlier studies on dislocation-related whisker growth in AgBr^31^. Hence, compact whisker electrodes could be the potential key for high-rate-capable lithium-metal anodes^28^. Reducing the brittleness of solid electrolytes by introducing dislocations as demonstrated for, e.g., perovskite SrTiO_3_^32^, could significantly reduce the ability of crack formation during Li plating, a prerequisite to enable ultra-high-plating rates^33^.

Despite the high potential of dislocations to overcome current obstacles in developing SSLB, a discussion in this context is absent. This is related to the difficulty to introduce dislocations into ceramics and the associated need for detailed understanding in mechanics. In contrast to metals, the mobility of dislocations in ceramics is limited due to their ionic or covalent nature of bonds. Nevertheless, several exceptions, such as LiF single crystals are known to be deformable even at room temperature^34–36^. Deforming polycrystals, however, requires at least five mobile slip systems^36,37^, which are not available at room temperature^38^. Since an increase in temperature activates additional slip planes a dislocation-based plastic deformation of polycrystalline ceramics becomes feasible^39–41^. Nevertheless, making a conclusion whether a certain ceramic can be successfully deformed by dislocation motion remains in particular challenging, since each crystal structure shows unique mechanical behavior with substantial variations even among different compositions in combination with the multitude of competing mechanisms that can operate simultaneously. Hence, a fundamental effort is needed to identify strategies to introduce dislocations into ceramic electrolytes to unroll their potential for future applications.

In order to test the feasibility to introduce dislocations by plastic deformation, we use Li_6.4_La_3_Zr_1.4_Ta_0.6_O_12_ (LLZTO) garnets as a model system to evaluate the plastic deformability of solid electrolytes. Based on the recorded stress–strain curves of LLZTO in combination with a transmission electron microscopy (TEM) analysis clear evidence is found for plastic deformation. While a final judgement of the dominating process remains challenging, key bottlenecks on the route to using dislocations in solid electrolytes are identified. These results can be considered as a new avenue in order to design interfaces in solid-state Li batteries that avoid Li nucleation/penetration by surface toughened LLZO and the formation of Li-whisker electrodes that enables high power batteries.

## Experimental

### Sample preparation

Hot-pressed Li_6.4_La_3_Zr_1.4_Ta_0.6_O_12_ (p-LLZTO, Ø 1 cm, 2 mm thickness) were purchased by Toshima Manufacturing. A Li_6_La_3_ZrTaO_12_ (LLZTO) single crystal was grown using the Czochralski pulling technique. Composition of the starting melt was stoichiometric with an additional excess of Li_2_O of 20 mol%. The raw materials, Li_2_CO_3_, La_2_O_3_, ZrO_2_, and Ta_2_O_5_ were weighed, mixed, and pressed isostatically at 2 kbar, sintered for 6 h at 850 °C, ground, pressed again, and sintered for 6 h at 1230 °C. For the growth process, this starting material was melted in an inductively heated, 40 ml iridium crucible enclosed by alumina ceramic insulation in a pure N_2_ ambient. After melt homogenization a thick iridium wire was immersed in the melt to initiate crystallization. With some material attached, the wire was slowly pulled upwards (0.5 mm h^−1^) and the generator controlled by the automatic diameter control routine of the pulling station. After the growth was completed, the crystal was withdrawn from the melt and cooled down to room temperature in 15 h. The obtained crystal had a length of 40 mm at a diameter of 15 mm. Its upper, first grown part was severely damaged due to cracking and spalling. But the lower half of the crystal boule was of good quality, colorless and mainly transparent, with only very few cracks near the surface. Composition of the crystal was tested for several points on a slice cut perpendicular to the growth direction by X-ray fluorescence analysis using a Bruker TORNADO M4 spectrometer. All measured elements (La, Zr, Ta) showed an even distribution over the crystal’s cross section with [Ta]:[Zr] ≈ 1 meaning that during crystallization the Ta distribution coefficient is nearly 1. Details about its structural and electrical properties can be found in, e.g., Ref.^23,24,42^. Single crystals were compressed along a [150] direction which allows a maximum Schmid factor of 0.38 for the <111> {1–10} slip system. The influence of crystal orientation was excluded by cross-check experiments with a compression axis tilted by 45°. Testing in these two directions makes sure that any slip system is oriented in with a Schmid factor > 0 in at least one experiment. All samples were shaped into approximately 2 mm × 2 mm × 4 mm cuboids.

### Plastic deformation

For deformation, a load frame (Z010, Zwick GmbH & Co. KG, Ulm, Germany) equipped with centered alumina rods for sample contacting, was used. Those were surrounded by a clamshell furnace (LK/ SHC 1500-85-150-1-V-Sonder, HTM Reetz GmbH, Berlin, Germany). The sample displacement was quantified with a linear variable differential transducer system with a nominal accuracy of 10 nm and final height reduction of the sample selectively confirmed ex-situ with a µm gauge.

The chosen temperature range between 980 and 1150 °C was determined by the operation limit for superalloys^43^ that could be used as forming tools for high-temperature shaping of polycrystalline ceramics. In order to obtain a deformation map with some predictability for other stresses and strain rates, a large spread of strain rates was aspired with applied compressive stress levels ranging from 5 to 100 MPa. As a result, strain rates down to below 1 × 10^–6^ s^−1^ (which approaches the instrumentation limit) and up to above 1 × 10^–3^ s^−1^ could be obtained. All tests were done with a heating rate of 5 K/min and a pre-load of 1.5 MPa. The temperature was equilibrated for 20 min when reaching the experiment’s temperature. Samples were cooled to room temperature several minutes after removing the load. Only compressive stresses were applied which are given as absolute values throughout the manuscript.

### TEM characterization

TEM sample preparation was performed via focused ion beam (FIB) milling with Ga ions using a FIB/SEM Dual Beam Microscope FEI NOVA 200. The lift out and initial milling step was done with 30 kV ions and the final milling step was performed at 5 kV. The resulting lamellae were mounted onto an Omniprobe copper-based lift-out grid and directly transferred to the microscope. STEM observations were carried out by a probe corrected FEI TITAN^3^ G2 microscope operated at 300 kV in scanning mode. Selected area electron diffraction (SAED) experiments were performed using a FEI Tecnai 12 microscope, operated at 120 kV. TEM samples were prepared from both the surface as well as from the inside of the cuboids after careful cracking.

## Results and discussion

### Deformation under compressive stress

**Figure 1:**
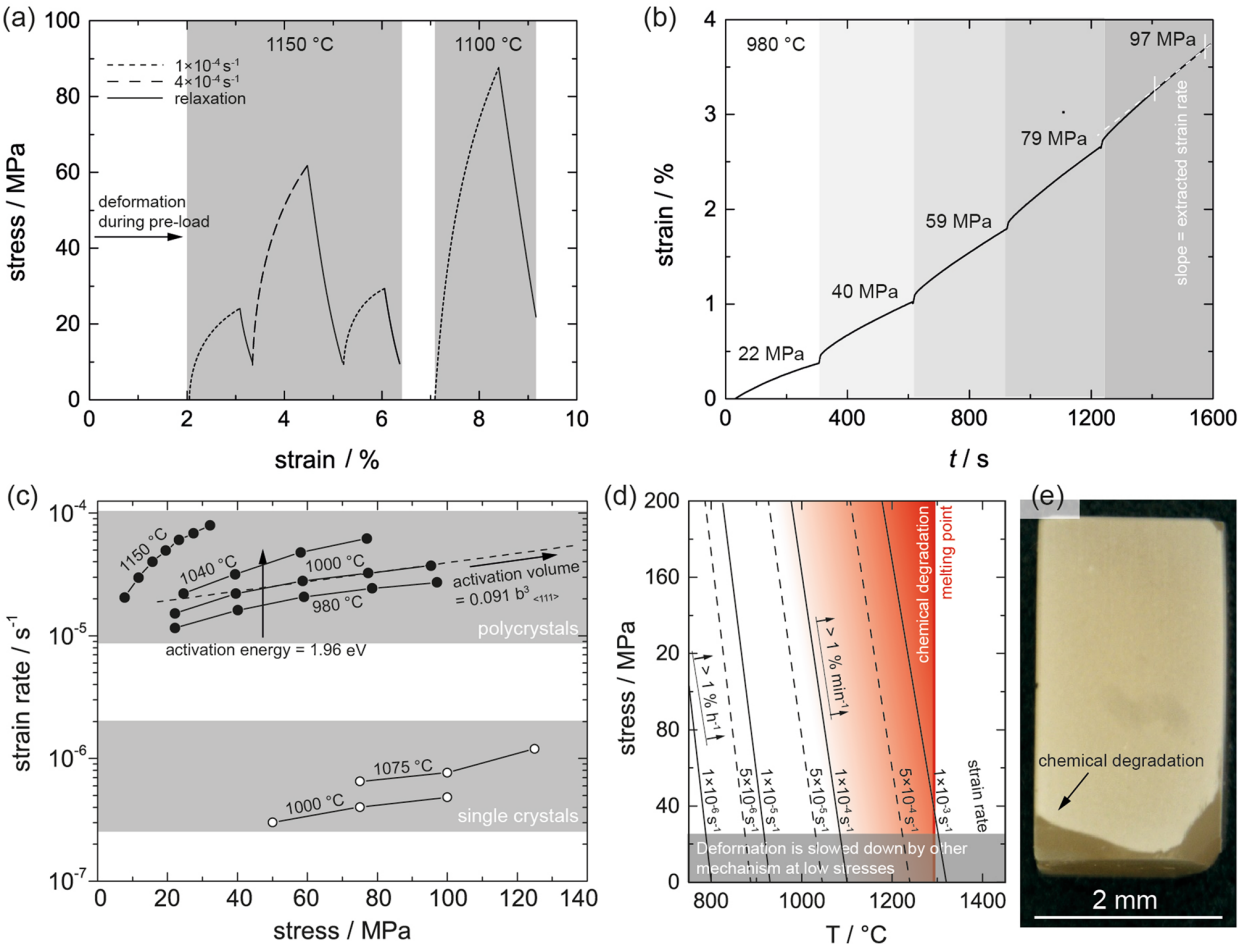
(**a**) Stress strain curves of LLZTO deformed at 1150 °C and 1100 °C. Multiple loading and deformations beyond 10% are possible at low stresses. Strain rate and temperature impact the deformation behavior significantly. (**b**) Strain in dependence of time measured in response to an applied stress during an experiment where stress is increased stepwise and held constant in between. The slope of the strain is extracted as the strain rate and used in (**c**). (**c**) Strain rate in dependence of stress as extracted from experiments demonstrated in Figure (**b**). In the range of 980 °C to 1040 °C polycrystalline LLZTO can be reliably deformed. At 1150 °C deformation is still possible, however, chemical degradation causes severe problems. Single crystalline LLZTO was observed to deform at much lower rates under comparable conditions. (**d**) Achievable strain rate determining the maximum possible deformation rate of polycrystalline LLZTO plotted as a function of applied stress and temperature. Chemical degradation occurs increasingly fast at temperatures just below the melting point, indicated in red shades. At too high stress, fracture can occur instead of deformation. At too low stress, however, deformation is retarded by other mechanisms. Consequently, a field of feasible processing parameters is defined and this graphic allows to identify the achievable strain rate immediately. (**e**) LLZTO polycrystal deformed by 5% at 1040 °C with a thin chemically degraded layer on the outside.

The plasticity of p-LLZO was demonstrated in compression tests at different strain rates as a function of temperature. Compressing p-LLZTO with a strain rate of 10^–4^ s^−1^ at 1150 °C results in a deformation of 1% relaxing again when the applied stress is released as shown in the stress–strain plot (Fig. 1a). Increasing the strain rate by a factor of four requires a significantly higher stress indicating a strong strain rate sensitivity. Similar observations are made by decreasing the temperature (e.g., 1100 °C) indicating a strong temperature dependence of the deformability. Overall, a maximum deformability of beyond 10% could be achieved. Despite of the large deformability of p-LLZO an elastic behavior and a yield point as typically observed in metals at room temperature are not identifiable. Such an absence of a pronounced yield point is a clear indicator that the mechanical behavior is not directly comparable to room temperature deformation.

In order to study the activation energy and the stress dependence (quantified by the activation volume), specific stress levels were applied stepwise for a certain period of time while recording the strain response (Fig. 1b).

The corresponding strain rates, $\dot{\epsilon}$, for particular temperatures over the stress, $\sigma$, applied are plotted in Fig. 1c, whereby the data points from 980 to 1040 °C were used to determine the activation energy and the stress dependence.

The dependence of the strain rate $\dot{\epsilon}$ on temperature, *T*, and $\sigma$, follows an Arrhenius law and can be expressed by Eq. (1).

1 $$\dot{\epsilon} = \dot{\epsilon}_{0} \cdot \exp \left( { - \frac{{E_{a} - V\left( \sigma \right) \cdot \sigma }}{kT}} \right).$$

It contains the microstructure and dislocation density dependent base strain rate, $\dot{\epsilon}_{0}$, the activation volume, *V*($\sigma$) the activation entalphy, *E*_a,_ and the Boltzmann constant, *k*. This phenomenological fitting law can be used for a variety of processes. Consistently inserting uniaxial stress $\sigma$ allows it to be used for diffusion as well while a conversion to a shear stress $\tau$ through the Schmid factor is preferred when discussing dislocation based processes only.

Based on the data points at 60 MPa and 80 MPa (see Fig. 1c) *E*_a_ and $\dot{\epsilon}_{0}$ were determined to be 1.96 eV and 1619, respectively.

The dependence of strain rate on the applied stress can be expressed in terms of the activation volume, *V*($\sigma$).

Multiplied with the stress, it reduces the effective activation energy (see Eq. 1) and can be determined from the presented data with Eq. (2)

2 $$V\left( \sigma \right) = kT\left[ {\frac{{\partial \ln \left( {\dot{\epsilon} \cdot 1s} \right)}}{\partial \sigma }} \right]_{T} = 1.38 \times 10^{ - 28} {\mathrm{m}}^{ - 3} ,$$

where the strain rate is multiplied by one second to make the argument dimensionless^41^.

The lattice constant of LLZTO is about 1.288 nm^42^ and the Burgers vector (measure for the displacement caused by one dislocation), is ½ <111> for the presumably most active slip system (< 111 > {-110} according to previous studies on natural abundant garnets or synthetic garnets, such as Y_3_Al_5_O_12_)^44–47^. The magnitude of the Burgers vectors |*b*_½ <111>_| is 1.123 nm. In consequence, the activation volume can be expressed as 0.097 *b*^3^.

The determined activation parameters allow us to replot and slightly extrapolate the data into a more intuitive presentation depicted in Fig. 1d. By re-arranging Eq. (1), iso-lines of specific strain rate levels $\dot{\epsilon}$ can be plotted in a diagram of stress and temperature using Eq. (3)

3 $$\sigma = \frac{{E_{a} + \ln \left( {\frac{{\dot{\epsilon}}}{{\dot{\epsilon}_{0} }}} \right) \cdot k \cdot T}}{V\left( \sigma \right)}$$

In addition, boundary conditions, such as the melting point, and the temperature when degradation starts to play a role, are indicated. Achievable strain rates are now immediately apparent for any combination of stress and temperature.

### Deformation mechanism

A pronounced deformation of p-LLZTO is clearly demonstrated. However, pinning down the dominating deformation mechanism is difficult because of competing processes taking place simultaneously, such as dislocation motion, diffusion creep or degradation. Note here, that the field of deformability of LLZTO is rather unchartered compared to well investigated properties such as conductivity. To identify the predominating process the analysis of the (1) activation energy, (2) activation volume, (3) diffusion creep, and (4) defect structure is required.

#### The activation energy

The fundamental propagation of dislocations occurs by the formation of a double-kink on the dislocation line which then expands sideways to advance the entire dislocation line by one Burgers vector^48,49^. This kink-formation is required for the motion of dislocations and, since the Burgers vectors are large in the garnet lattice, significant thermal activation is required to mobilize dislocations. Typical activation energy for a dislocation-based plastic deformation is below 0.5 eV for metals, and between 1 and 10 eV for ceramics^50–52^. For garnet single crystals, e.g., Y_3_Al_5_O_12_ and Ca_3_Ga_2_Ge_3_O_12_, values between 3.5 and 7 eV (or beyond^53^) at about 1750 °C are reported, respectively^45,54^. Here, an activation enthalpy of 1.96 eV was observed for p-LLZTO at about 1000 °C, which is significantly lower than observed for other garnets. Moreover, in polycrystalline garnets, activation energies for diffusion creep are similar to the activation energies for dislocation-based processes, which makes distinguishing between processes based on the activation energy difficult^55^.

In order to identify the dominating process, we attempt to set the activation energy of LLZTO into context, by assuming that (1) the diffusion rate (of the slowest diffusing species) is proportional to the homologous temperature (fraction of temperature/melting point) and, simultaneously, (2) the thermal activation of dislocation motion is determined by crystal structure, slip system and related to the absolute temperature. These simplifying assumptions are, e.g., evident for the activation enthalpy of 3 eV and 6 eV for the <100> {100} and <110> {110} slip system observed in SrTiO_3_, respectively^52^. Based on the made simplifications, we can now consider that, (1) the temperature required for dislocation motion is comparable among garnets, and (2) the sintering temperature (determined by the diffusion rate of the slowest diffusing species) scales with the melting point. For Y_3_Al_5_O_12_ with a melting point of 2000 °C it was shown that 1000 °C is insufficient to enable dislocation motion^44,56^. Hence, this perspective would suggest diffusion to prevail over dislocation motion^44,45^.

We also flip the perspective and compare the temperature needed to reach a strain rate of, e.g., 10^–6^ s^−1^ for different garnets. Again, this needs several simplifications out of which the following two are crudest: (1) The dislocation density in all samples was equal and (2) the strain rate is not a function of strain. This temperature was observed to be 1075 °C for LLZTO here while it was 1250 °C for Ca_3_Ga_2_Ge_3_O_12_ and 1700 °C for Y_3_Al_5_O_12_^45,54^. The large discrepancy between Ca_3_Ga_2_Ge_3_O_12_ and Y_3_Al_5_O_12_ suggests that LLZTO may fall in the pattern. On the other hand, it suggests that the simplifications of the previous perspective are inaccurate. In this context, a profound conclusion on the dominating process based on the activation energy is not possible at current state^39,41,57^.

#### The activation volume

Additionally, the predominating deformation process of materials can be identified by using the activation volume (i.e., a fitting parameter which allows to describe the dependence of the effective activation energy of the process on applied stress)^41,52^. For example, in SrTiO_3_^51,58^ the deformation is rate limited by a kink-pair mechanism, which is associated to an activation volume of typically 1 b^3^^41^. For garnets such knowledge is, unfortunately, not available. Here, the activation volume was found to be only 0.1 b^3^ which is rather low and may be an indication for a process involving an even smaller number of atoms, e.g., diffusion.

#### The diffusion creep

A simple way to exclude a series of mechanisms, such as diffusion creep, is testing deformability on single crystals, as, e.g., grain boundaries are absent^44,45,54^. However, single crystals are often produced with an extremely low dislocation density^59^ while simultaneously the dislocation velocity can be in the range of only nm s^−1^^60^. When dislocation-free crystal volumes (often with the size of even 1 mm^3^) exist, it can take hours for the dislocation density to sufficiently increase in order to accommodate higher strain rates. This can cause a severe delay time (even in the range of hours) before yield occurs^40,53,61^. In Fig. 1c, we also present strain rate data for single crystals which shows significantly slower deformation. With grain boundaries as dislocation sources at high temperatures, polycrystals can adjust their dislocation density much faster than single crystals, reducing delay times, which could explain the higher strain rates observed for p-LLZTO. However, degradation of the single crystals sets in before a possible delay time for the yield is overcome^61^. Hence, the compression experiments of single crystals do not give evidence for or against a high dislocation mobility.

In order to further pinpoint that a dislocation driven mechanism is responsible for the observed deformation the grain size dependency of diffusion can be analyzed. However, the synthesis of LLZTO samples with different grain sizes is challenging, due to the high sensitivity of the sample quality on the process parameter (e.g., *T*, dwelling time). Small contamination layer on the grain boundary caused by, e.g., Li-loss or segregation of dopant elements in grain boundaries can allow grain boundary sliding^62^ and early sample failure. Only after substantial rework on the synthesis^63–65^ successful dislocation based deformation is attained^32^.

#### The defect structure

**Figure 2:**
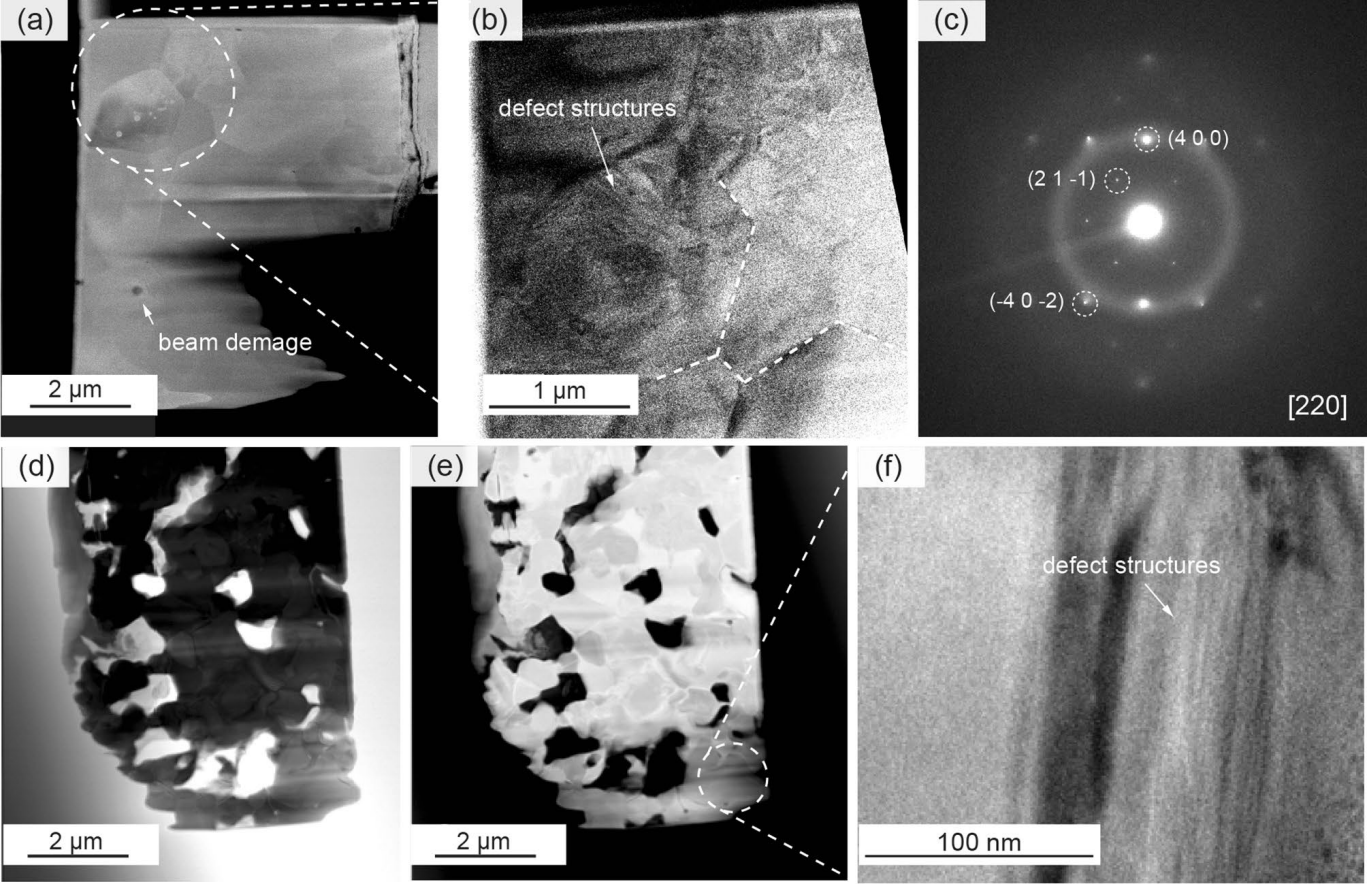
(**a**) STEM annular dark-field (ADF) image from a FIB lamella taken from a region inside of the bulk showing the micro structure of the material with grain sizes in the range of 0.2–1.5 µm. The material shows high beam sensitivity. (**b**) Contrast variations in bright field (BF) mode within the grains hint towards the presence of extended defect structures and dislocations in (**a**). (**c**) The SAED diffraction pattern taken from the grain interior of grains shown in (**b**) can be assigned to the garnet structure (SG *I*-*a*3*d*, no. 230). Significant surface amorphizations is observed as indicated by halo rings. (**d**, **e**) STEM image of FIB lamella prepared form the degraded surface layer showing compositional differences between the grains but high beam stability (ADF (**d**), BF (**e**)). (**f**) Defect structures are found in areas of low lamella thickness in (**d**, **e**).

FIB lamellas were prepared from the interior and surface of p-LLZTO as shown in Fig. 2a, d, and e respectively. The grain size can be determined to be in the range between about 0.3 to 1.3 µm (Fig. 2b). In contrast to the lamella prepared from the interior the lamella taken from the surface shows significant degradation (see also Fig. 1e). This degradation can be assigned to the formation of La_2_O_3_ and La_2_Zr_2_O_7_, which are typical phases formed due to Li-loss during a high temperature treatment^66^. The defect structures are clearly seen in both, the surface (Fig. 2f) and the interior (Fig. 2b), however, a conclusive judgement of the type of theses defect structure is not straightforward.

The complexity is related to the challenges on TEM based characterization techniques, such as the (1) air and water sensitivity of LLZTO that requires the use of water free solvents for all sample preparation steps as well as the sample transfer from preparation to the electron microscope^67^ without exposure to ambient air, and (2) high sensitivity of LLZTO to the electron beam as it degrades quickly under electron beam irradiation (see Fig. 2a), which prohibits dose extensive characterization techniques such as high-resolution imaging. Moreover, significant surface amorphization can be seen in the selected area electron diffraction (SAED) provided in Fig. 2c, which was acquired from a single grain (Fig. 2b). The pattern shows a distinct halo ring that is characteristic for the presence of amorphization. This amorphous surface layer is introduced most likely by the final ion milling step during sample preparation or subsequent exposure to ambient conditions during sample transfer and is particularly seen in the images at low thickness regions, where the contrast between individual grains starts to vanish. The spots in the pattern can be assigned according to the [220] crystallographic orientation of cubic LLZTO with SG *Ia*-3*d* (*no.* 230) of the grain with regard to the electron beam.

To overcome these limitations to reach an equal image quality as reported for, e.g., air stable SrTiO_3_ polycrystals^32^ substantially more effort is needed. For example, using a cryo sample holder, the use of lower acceleration voltages, low-dose techniques, and a gentle sample preparation including a transfer without exposure to ambient conditions. Nevertheless, similar defect structure appeared and were identified as dislocations in ceramics with other crystal structure previously by Kim et al.^68^ and Ren et al.^69^, which gives further indications for their successful incorporation in LLZTO by plastic deformation.

## Conclusions

Based on the recorded stress–strain curves of LLZTO we found clear evidence for plastic deformation up to 10 %. Careful mechanical analysis of polycrystalline and single crystalline samples allows identifying a process window. Quantification of activation energies and other crucial parameters gives first insight into the dislocation-based deformability as a first step to fully understand the mechanical complexity involved. While extended analysis is required for providing a complete picture, our preliminary TEM study is giving additional hints that suggest that the introduction of dislocations into LLZTO may be feasible. A final proof remains, however, challenging, due to competing processes potentially taking place simultaneously, such as dislocation motion, diffusion creep, and degradation at high temperatures, as well as the high electron beam-sensitive of LLZTO. Hence, new characterization strategies need to be developed to study dislocation in ceramic electrolytes. For example, a systematic study on the deformability as a function of the amount of dislocation sources controlled by tuning the amount of grain boundaries could be performed. Moreover, alternative strategies to introduce dislocation directly at the interface, at lower temperatures, and without applying high uni-axial pressure (e.g., polishing^70–72^) can be considered.

In summary, this manuscript considers for the first time dislocation as a potential new strategy to design interfaces in SSLB that avoid Li metal nucleation/penetration by surface toughened LLZO and the formation Li-whisker electrodes. Despite of the absence of a final proof our preliminary discussion on the underlying mechanisms related to plastic deformation of LLZTO provides a feasible process window for deformation, that is a key step on the road of making dislocations available as a design element for solid state batteries.
